# Comparative analysis of effectiveness for phage cocktail development against multiple *Salmonella* serovars and its biofilm control activity

**DOI:** 10.1038/s41598-023-40228-z

**Published:** 2023-08-11

**Authors:** Jhonatan Macedo Ribeiro, Giovana Nicolete Pereira, Itamar Durli Junior, Gustavo Manoel Teixeira, Mariana Marques Bertozzi, Waldiceu A. Verri, Renata Katsuko Takayama Kobayashi, Gerson Nakazato

**Affiliations:** 1 Laboratory of Basic and Applied Bacteriology, State University of Londrina, Londrina, PR Brazil; 2 Laboratory of Bioinformatics, Federal University of Santa Catarina, Florianópolis, SC Brazil; 3 Laboratory of Microbe Biotechnology, State University of Londrina, Londrina, PR Brazil; 4 Laboratory of Pain, Inflammation, Neuropathy, and Cancer, State University of Londrina, Londrina, PR Brazil

**Keywords:** Bacteriophages, Biofilms

## Abstract

Foodborne diseases are a major challenge in the global food industry, especially those caused by multidrug-resistant (MDR) bacteria. Bacteria capable of biofilm formation, in addition to MDR strains, reduce the treatment efficacy, posing a significant threat to bacterial control. Bacteriophages, which are viruses that infect and kill bacteria, are considered a promising alternative in combating MDR bacteria, both in human medicine and animal production. Phage cocktails, comprising multiple phages, are commonly employed to broaden the host range and prevent or delay the development of phage resistance. There are numerous techniques and protocols available to evaluate the lytic activity of bacteriophages, with the most commonly used methods being Spot Test Assays, Efficiency of Plating (EOP), and infection assays in liquid culture. However, there is currently no standardization for which analyses should be employed and the possible differences among them in order to precisely determine the host range of phages and the composition of a cocktail. A preliminary selection using the Spot Test Assay resulted in four phages for subsequent evaluation against a panel of 36 *Salmonella* isolates of numerous serovars. Comparing EOP and infection assays in liquid culture revealed that EOP could underestimate the lytic activity of phages, directly influencing phage cocktail development. Moreover, the phage cocktail containing the four selected phages was able to control or remove biofilms formed by 66% (23/35) of the isolates, including those exhibiting low susceptibility to phages, according to EOP. Phages were characterized genomically, revealing the absence of genes associated with antibiotic resistance, virulence factors, or integrases. According to confocal laser scanning microscopy analysis, the biofilm maturation of one *Salmonella* isolate, which exhibited high susceptibility to phages in liquid culture and 96-well plates biofilm viability assays but had low values for EOP, was found to be inhibited and controlled by the phage cocktail. These observations indicate that phages could control and remove *Salmonella* biofilms throughout their growth and maturation process, despite their low EOP values. Moreover, using infection assays in liquid culture enables a more precise study of phage interactions for cocktail design timelessly and effortlessly. Hence, integrating strategies and techniques to comprehensively assess the host range and lytic activity of bacteriophages under different conditions can demonstrate more accurately the antibacterial potential of phage cocktails.

## Introduction

*Salmonella* spp. are a frequent human pathogen recognized worldwide as a public health threat^1^. Among foodborne pathogens, *Salmonella* is ranked as the third leading cause of death and, therefore, also represents a significant economic burden. The latest World Health Organization report on the global burden of foodborne diseases estimated that of 88 million annual cases caused by *Salmonella* worldwide, 123,000 resulted in death, and a sum of 222,000 years of life were lived with disability^1^.

One of the main characteristics of *Salmonella* spp. that allows its survival in hostile environments is its formation of surface-associated complex communities–biofilms–that makes it difficult to control its proliferation, especially in poultry farms, since more than 50% of *Salmonella* strains isolated from chickens slaughtered in Brazil demonstrate the ability to form biofilms^2–5^. Biofilms increase tolerance to biocides^6,7^, given the organization of cells within the polymer matrix, which reduces penetration of the biocidal agent and causes the bacteria to persist in food processing environments for long periods^5,8^.

Therefore, it is of great importance to develop strategies to control *Salmonella* in poultry production, aiming to reduce the use of antibiotics due to the emergence of multidrug-resistant microorganisms^9,10^. In this scenario, bacteriophages are gaining interest as agents for inhibiting or disrupting biofilms^11–13^.

Initially discovered by Frederick Twort and Felix D’Herelle, bacteriophages (phages) are viruses that specifically infect bacteria^14,15^. As they can trigger the lysis of their hosts at the end of multiplication, lytic phages have been used in medical practice in the East countries such as Poland and Georgia since their discovery, replacing conventional antibiotics or in combination with them^11,14^. Numerous studies have documented phages as a helpful tool for the inactivation and control of foodborne pathogens^14,16–18^, which allowed the phage-based products against *Salmonella* spp. to be approved as Generally Recognized as Safe (GRAS) by the U.S Food and Drug Administration (FDA)^13,16,19^.

Phages can be applied as biological control either as a single phage type (monophage) or in a cocktail comprising several phages^20,21^. By combining several phages, it is possible to increase the spectrum of action toward the number of strains that phages can infect, as well as to avoid bacterial resistance to phages, which can quickly occur in cases where only one phage is used^11,20^. Therefore, it is necessary to investigate the host range and the virulence of the phages that constitute the cocktail to ensure its effectiveness^11,22,23^.

Even though there are no defined guidelines to standardize the development of an optimized cocktail, some techniques and protocols such as PhageScore^24^, Virulence Index^11,23^, and Breadth and Depth of the cocktail^21^ have been developed to quantify phage efficiency and determine the best approach for cocktail design. Therefore, this study demonstrates the characterization of four phages, evaluating the potential of a proposed cocktail for the biological control of *Salmonella* spp. Furthermore, the proposed experiments provide quantitative data to assess the virulence of phages, their ability to disrupt biofilms, as well as the safety of the phages concerning the lytic cycle, bacterial virulence genes, and resistance genes.

## Results

### Combination of methods for screening and selection of *Salmonella* phages with high lytic activity

A total of 24 phages were first isolated for 11 *Salmonella* isolates out of 50 (Supplementary Table S1) used for enrichment. The phages were selected based on the morphology of the plaques (plaque size and turbidity), varying from 0.3 to 1.1 mm in diameter, and the host used for isolation, i.e., plaques from distinct hosts were considered different phages. However, as discussed later, the same phage could exhibit different plaque morphologies for different bacterial isolates.

Four phages out of the 24 first isolated phages were selected for further characterization as they exhibited a broad host range for 50 *Salmonella* isolates comprising 16 distinct serotypes (Supplementary Fig. S1) in Streak Spot Test (SST) assay. Based on phages' phylogenetic classification, they were named *Tequintavirus* phA11, *Tequintavirus* phC11, *Tequintavirus* phB7, and *Tequintavirus* phC17, and from now on, they will be called just phA11, phC11, phB7, and phC17. Results of the SST assay (Supplementary Fig. S1) demonstrated a broad initial host range for the four selected phages with lytic activity percentages, i.e., the percentage of bacteria which presented a growth inhibition when in contact with the phage suspension, ranging from 50% to phA11, 58% to phC11, 66% to phB7, and 66% to phC17.

**Figure 1:**
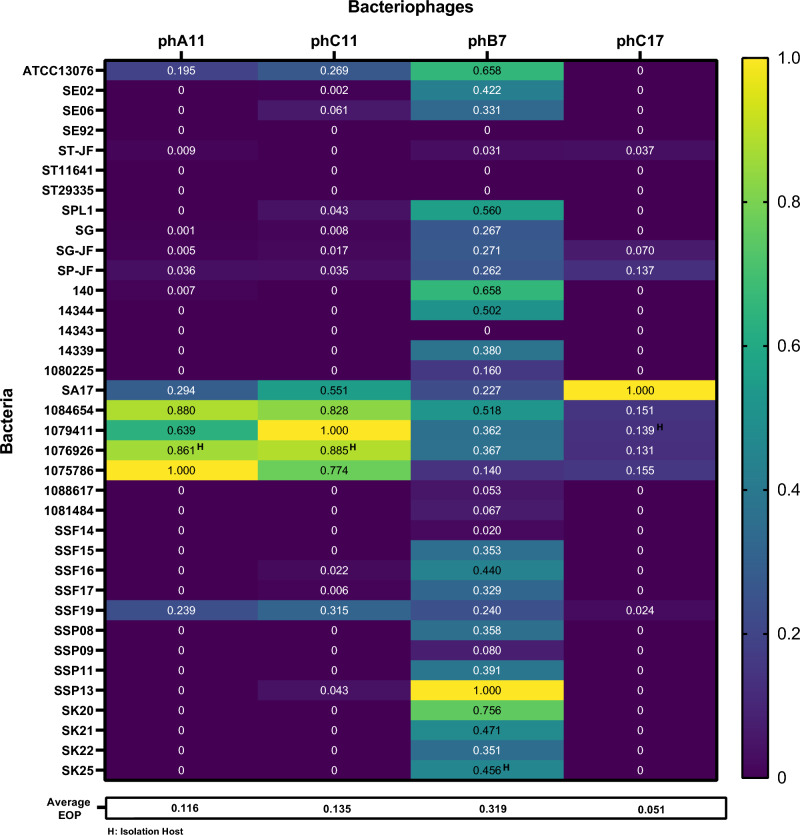
Heatmap of relative Efficiency of Plating for phages phA11, phC11, phB7, and phC17 for 36 *Salmonella* isolates. Values represent the average of three replicates. The EOP value for the phage-bacteria combination was classified as “High” for ratios ≥ 0.5, “Medium” for ratios ≥ 0.1 and < 0.5, “Low” for values > 0.001 and < 0.1, and “Inefficient” for ratios ≤ 0.001.

**Figure 2:**
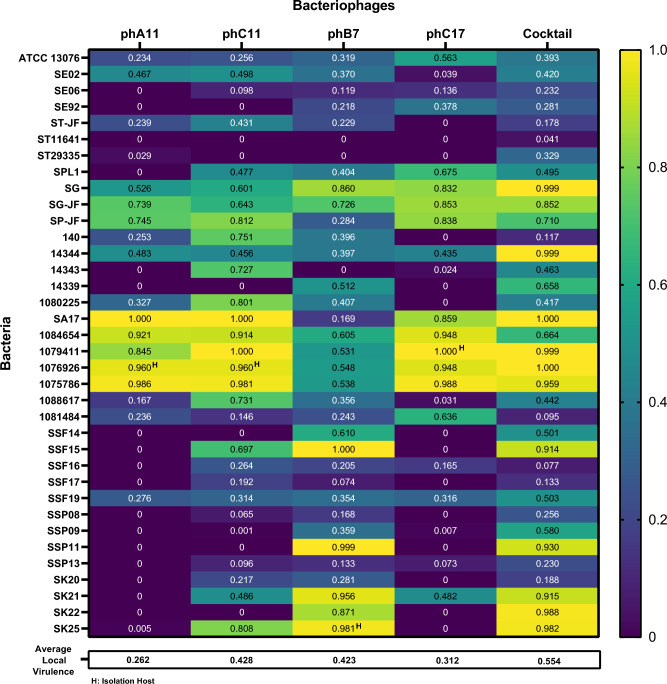
Heatmap of Local Virulence of MOI 1 (*v*_1_) for phages phA11, phC11, phB7, phC17, and phage cocktail for 36 *Salmonella* isolates. Values represent the average of three replicates. Growth curves are shown in Supplementary Fig. S2. Local Virulence score was classified as “High” for *v*_1_ > 0.5, “Medium” for 0.2 ≤ *v*_1_ ≤ 0.5, “Low” for 0.001 ≤ *v*_1_ < 0.2, and “Inefficient” for *v*_1_ < 0.001.

The 36 sensitive isolates in the SST assay were used for plaque formation evaluation by the relative Efficiency of Plating (EOP) method (Fig. 1) and to evaluate the virulence of the phages by Local Virulence at Multiplicity of Infection (MOI) 1 (*v*_1_) analysis (Fig. 2). EOP evaluates the ability of phage to form plaques by comparing the number of plaques that a phage is able to form in a given bacterial strain/isolate with the highest plaque number obtained for that phage among the tested bacteria group. Therefore, the bacterial strain/isolate with the highest plaque number formed for a specific phage was used as a reference to that phage and consequently had an EOP value of 1.0. Each phage formed the highest plaque number in different bacterial isolates: for phA11, it was the isolate 1075786; for phC11, it was the isolate 1079411; for phB7, it was the isolate SSP13; and for phC17, it was the isolate SA17. Moreover, all four phages formed the highest plaque number in bacterial isolates different from the ones from which they were isolated. Regarding *v*_1_, it can be defined as the capacity of phage to inhibit the growth of a bacteria in liquid culture at MOI 1. There was a difference in the host range obtained in each method and the classification of the virulence of the phages and cocktail. Table 1 describes the classification of the phages in each method, emphasizing the phB7 that, according to *v*_1_, presented high lytic activity against 36.1% of the isolates, while for EOP, 19.4% of the isolates had high production of plaques. For phC11, the same characteristic was observed, where it presented high lytic activity in *v*_1_ against 39.9% of the isolates, while for EOP, only 13.9% of the isolates had high production of plaques. Phage phC11 has an average *v*_1_ and EOP of 0.428 and 0.135, respectively, while for phage phB7, *v*_1_ and EOP average are 0.423 and 0.319, respectively. Differences among *v*_1_ and EOP values for phC11 and phC17 were intensified in the “Inefficient” classification, with *v*_1_ percentages of 19.4% and 38.9% and EOP percentages of 55.6% and 75.0%, respectively. Nevertheless, the cocktail had no strains in the “Inefficient” classification, considering *v*_1_ and high lytic activity against 50% of all tested isolates. For some bacteria, the cocktail presented positive interactions. Strain 14344 treated with phages phA11, phC11, phB7, and phC17 presented *v*_1_ scores of 0.483, 0.456, 0.397, and 0.435, respectively (Fig. 2). Conversely, the *v*_1_ score of the cocktail increased to 0.999 (Fig. 2). Analogous patterns were observed for isolates SSF19 and SSP09 (Fig. 2).

**Table 1:** Summary of EOP and *v*_1_ scores and classification of lytic activity of phages phA11, phC11, phB7, phC17, and phage cocktail.

Classification	Index	phA11 (%)	phC11 (%)	phB7 (%)	phC17 (%)	Cocktail
High	*v*_1_	22.2	38.9	36.1	30.6	50.0%
	EOP	11.1	13.9	19.4	2.8	N/A
Medium	*v*_1_	22.2	25.0	41.7	11.1	30.6%
	EOP	5.6	5.6	50.0	0.0	N/A
Low	*v*_1_	8.3	16.7	13.9	19.4	19.4%
	EOP	16.7	25.0	19.4	22.2	N/A
Inefficient	*v*_1_	47.2	19.4	8.3	38.9	0.0%
	EOP	66.7	55.6	11.1	75.0	N/A

**Figure 3:**
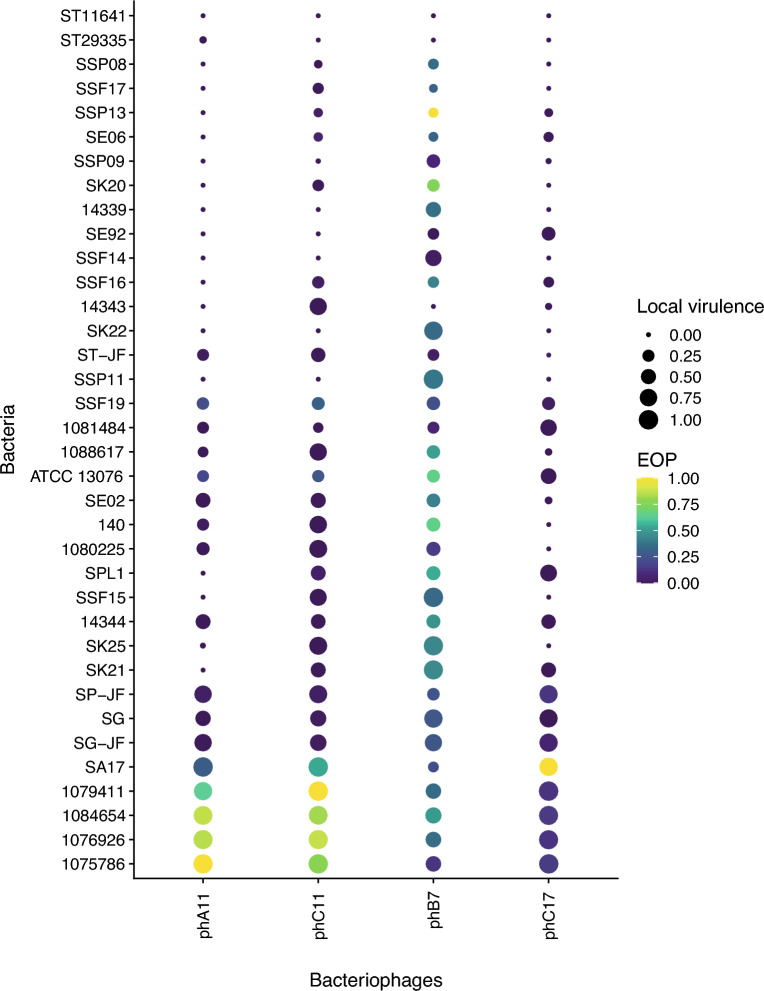
Dotplot for comparison of Local Virulence and relative Efficiency of Plating values for phages phA11, phC11, phB7, and phC17. The size of the dot at the intersection of Bacteriophages/Bacteria is proportionate to the mean Local Virulence value. The scale color is proportionate to mean EOP values.

This difference between the results of the two tests can be better seen in Fig. 3, in which the results were plotted to compare each other on a dotplot figure. The color scale represents the EOP values and the dot size represents the *v*_1_ values for each phage-bacteria combination. Data are ordered according to the highest mean *v*_1_ of the four phages to the lowest values from above to up. Phages phA11, phC11, and phC17 demonstrate a similar pattern of dots, although phC11 had the capacity to control the growth of some isolates that phA11 and phC17 could not. From 36 *Salmonella* isolates, only 5 (14%) demonstrate a direct correlation of phage susceptibility between EOP and *v*_1_ for phages phA11 and phC11. For phage phC17, this difference is even bigger, and only 1 (3%) isolate demonstrates the correlation between phage susceptibility tests. On the other side, phB7 presents a good capacity to form plaques, which can be seen as a general correlation of susceptibility between EOP and *v*_1_ once the color scale and dot size are more homogeneously distributed. Big dots (high *v*_1_ values) with purple color (low EOP values) can be seen for all phages except phB7, as a demonstration of differences of phage lytic activity evaluation depending on the phage analyzed.

### Characterization of selected phages demonstrates high stability and production properties

Bacteriophages were characterized by their maximum production and resistance to different physicochemical conditions.

The four selected bacteriophages produced the highest progeny in different Multiplicities of Infection (MOI) (Supplementary Fig. S3), which was then used in the subsequent experiments for maximum phage production. Due to the use of 10^8^ CFU/mL (> 10^7^ CFU/mL) of host strain, we assume that 100% phage adsorption has occurred soon following phage addition^25^.

The stability of bacteriophages was studied at eight different temperatures (−20 °C, 4 °C, 25 °C, 30 °C, 37 °C, 50 °C, 70 °C, and 90 °C) and three different times of incubation (1 h, 24 h, and 7 days—except for 50 °C, 70 °C, and 90 °C) (Supplementary Fig. S4). Storage at 4 °C was considered the control for stability since it is the temperature used for storing bacteriophage stock. Our results demonstrate that all phages exhibit good stability in the temperatures tested, except for 70 °C and 90 °C. Phage phC17 was less stable at 30 °C and 37 °C in 24 h and 7 days, decreasing around 0.8 log compared to 1 h. Although the phages had titer reductions in 7 days of incubation, they generally demonstrated good stability. Upon exposure to a temperature of 50 °C for 1 h, all four phages exhibited considerable stability, with no reduction in phage quantities observed. However, upon extending the incubation period to 24 h, a reduction in phage concentrations became evident. Subsequently, the phages’ resistance was tested at a higher temperature of 90 °C, both for 1 h and 24 h. All four phages exhibited a remarkable susceptibility to this elevated temperature, as their concentrations fell below the limit of detection, rendering them nonviable after exposure to 90 °C. At an intermediate temperature of 70 °C, the response of the four phages varied. Specifically, phage phB7 displayed a notable lack of resistance, showing a decrease in concentration following 1 h exposure as well as complete absence of plaques after 24 h of incubation. In contrast, the other three phages demonstrated varying degrees of resistance to 70 °C, as evidenced by log reductions in phage concentration after 1 h. Subsequently, following the 24 h incubation period, no plaques were detectable for these three phages, signifying their compromised viability at this temperature.

Additionally, the stability of phages was evaluated in a range of pH from 2.5 to 12.5 at the same times of incubation of the temperature stability experiment (Supplementary Fig. S5). All phages were inactivated at all time points at pH 2.5, and the concentration was under the detection limit. At pH 3.5, all phages remained active with 1 h of incubation, with a slight titer reduction. However, at 24 h of incubation, only phA11 remained active, decreasing around 1 log of viable phages compared to 1 h of incubation. None of the phages remained active within 7 days of incubation at pH 3.5. All phages were detectable in all time points for the remaining pH points, except for phages phC11 and phB7 at pH 12.5.

### Bacteriophage cocktail successfully controls the biofilm of multiple *Salmonella* serovars

**Figure 4:**
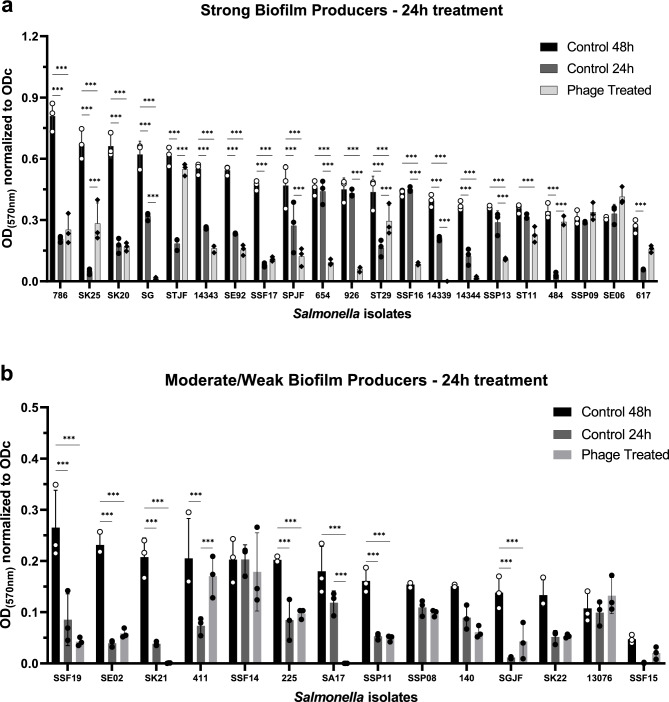
MTT biofilm assay data for phage cocktail. (**a**), *Salmonella* isolates classified as strong biofilm producers treated with phage cocktail for 24 h. (**b**), *Salmonella* isolates classified as moderate or weak biofilm producers treated with phage cocktail for 24 h. All data are presented as the mean OD_(570nm)_ normalized to the optical density cutoff (ODc) of three replicates with standard deviation bars for all bacterial isolates. Individual data points are indicated with dots. ***indicate a statistically significant difference (*p* < 0.001, two-way ANOVA and post hoc Tukey’s multiple comparison test). For graph aesthetics improvement, some identifications of bacterial samples were reduced to the last three numbers. Therefore, where it reads 786, read 1075786; 654, read 1084654; 926, read 1076926; 484, read 1081484; 617, read 1088617; 411, read 1079411; 225, read 1080225.

Thirty-five *Salmonella* isolates comprising 14 different serovars were used to evaluate the anti-biofilm activity of the phage cocktail. A phage cocktail composed of four phages at a final concentration of 5 × 10^7^ PFU/mL of each phage was used to treat for 24 h the biofilms. The results demonstrated a good capacity of biofilm controlling or removal (Fig. 4). The cocktail was able to control the biofilm growth of strong (Fig. 4a) and moderate/weak (Fig. 4b) biofilm producers, controlling or removing the biofilm of 23 (66%) out of 35 isolates tested (*p* < 0.05, two-way ANOVA and post hoc Tukey’s multiple comparison test). There was no detection of sessile cells after phage treatment for isolates 14339, SK21, and SA17, indicating the complete removal of biofilm. A significant reduction of OD values of phage treated compared to 24 h biofilm control condition (*p* < 0.05, two-way ANOVA and post hoc Tukey’s multiple comparison test) can be observed for isolates SG, SPJF, 654, 926, SSF16, and SSP13, indicating that although bacterial cells were not eradicated, phage cocktail could reduce the biofilm with 24 h of treatment. For 14 isolates, the phage cocktail was able to control the biofilm growth, maintaining the biofilm for 24 h at the same level as when the cocktail was applied or slowing down the biofilm growth, a fact observed by significant differences (*p *< 0.05, two-way ANOVA and post hoc Tukey’s multiple comparison test) of phage treated condition compared to 48 h biofilm control but no difference or slightly increase (*p* < 0.05, two-way ANOVA and post hoc Tukey’s multiple comparison test) when compared to 24 h biofilm control. No significant biofilm increase (*p* > 0.05, two-way ANOVA and post hoc Tukey’s multiple comparison test) was observed for phage treated compared to 48 h biofilm control, indicating that the phages in the cocktail did not increase biofilms' growth for any tested isolates.

**Figure 5:**
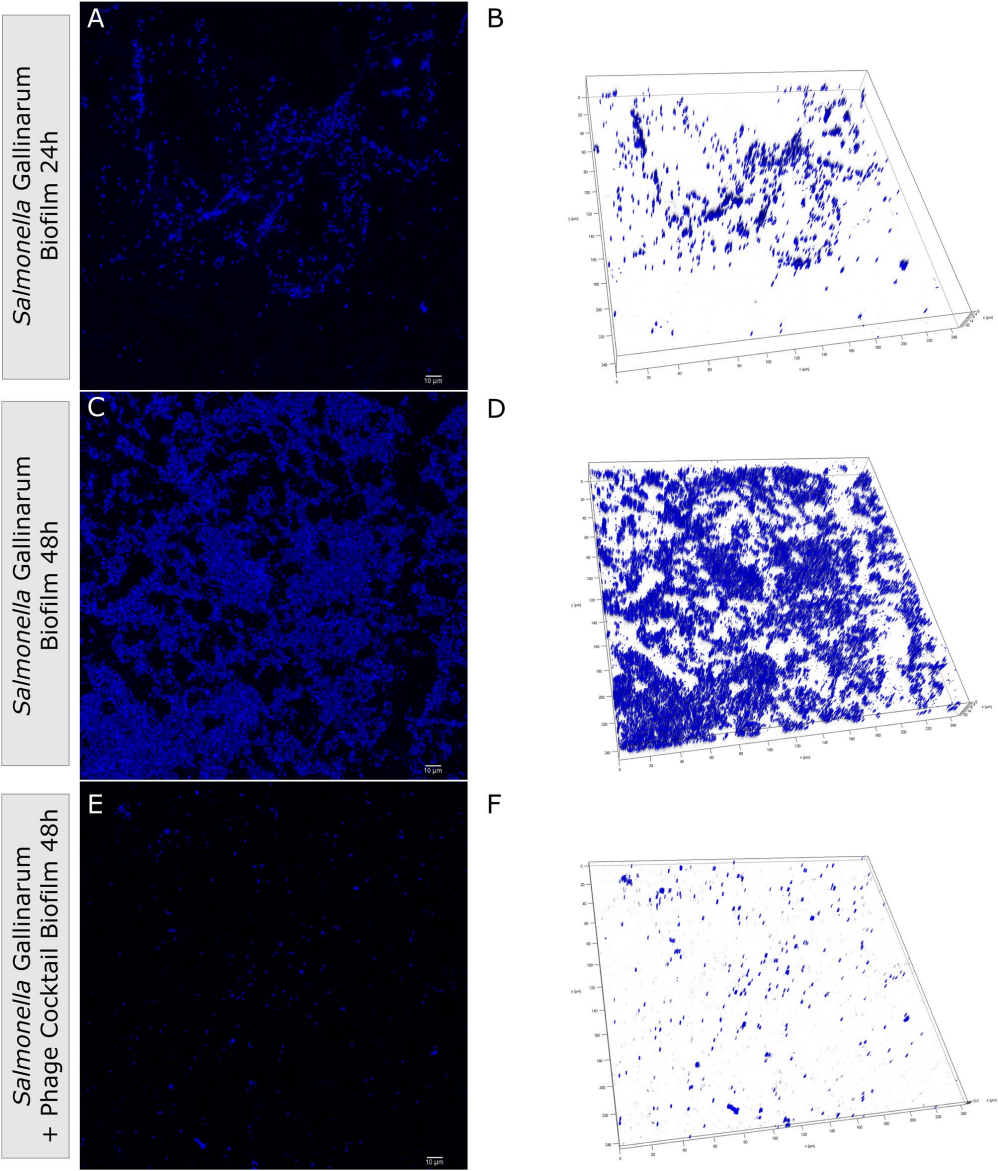
Representative CLSM images of DAPI stained *Salmonella enterica* serovar Gallinarum SG 24 h and 48 h biofilms at different conditions. SG cells were grown at 37 °C for 24 h (**a** and **b**) and 48 h (**c** and **d**) without phage cocktail addition for biofilm growth. A phage cocktail (2 × 10^8^ PFU/mL of all phages) was added to 24-h-old biofilms and incubated for 24 h (**e** and **f**). Scale bars represent 10 µm.

**Figure 6:**
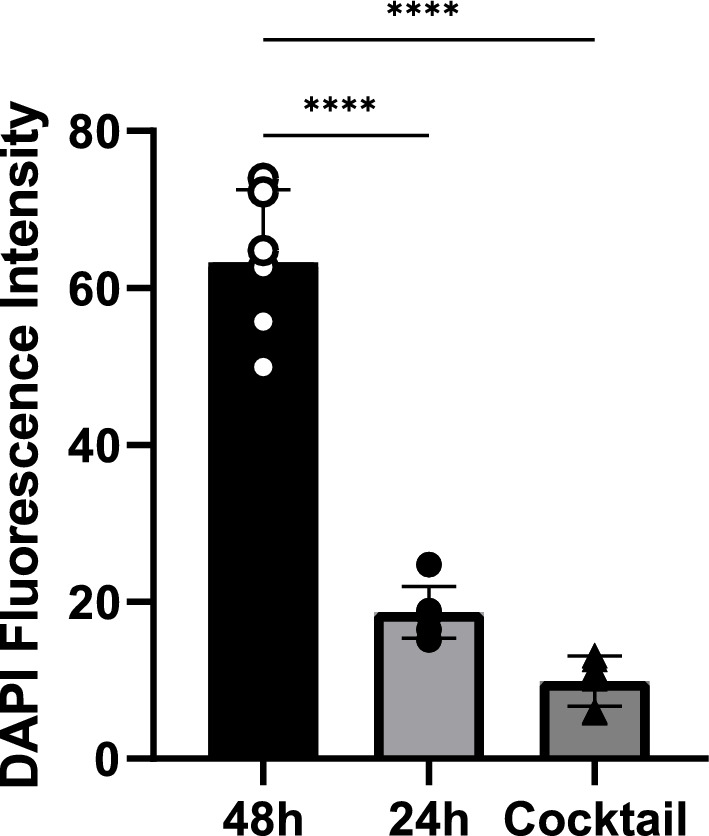
DAPI fluorescence intensity of CLSM images of *Salmonella enterica* serovar Gallinarum SG biofilms. Data are presented as the mean of six images of different fields with standard deviation bars. Individual data points are indicated with dots or triangles. ****indicate a statistically significant difference (*p* < 0.0001, one-way ANOVA and post hoc Tukey’s multiple comparison test) among groups.

Based on these results, *Salmonella* SG was selected for further analysis of phage interactions with biofilm since it presented considerable differences between the three conditions tested. A comparative analysis of controls and phage-treated biofilms was done on Confocal Laser Scanning Microscopy (CLSM) to better understand the effects of the phages on biofilm structure. Six images were taken, and one was chosen to represent each condition. After 24 h of incubation, biofilm control presented a thickness of around 20 µm (Fig. 5a and b). Biofilm control of 48 h presented a well-structured biofilm and thickness of around 20 µm but with higher cell density (Fig. 5c and d) when compared to 24 h control. Biofilm thickness (4 µm) and cell density were reduced after phage cocktail treatment (Fig. 5e and f) for 24 h. For quantitative analysis and comparison of the images, DAPI (a fluorescent DNA marker) fluorescence intensity was quantitated (Fig. 6) and showed similar patterns as demonstrated in the MTT biofilm assay, reinforcing the biofilm growth control activity of the phage cocktail against this bacterial isolate. The phage-treated condition presented, on average, a significant reduction (*p* < 0.0001, one-way ANOVA and post hoc Tukey’s multiple comparison test) of around seven-fold in fluorescence intensity compared to 48 h biofilm control. No significant differences (*p* = 0.0565, one-way ANOVA and post hoc Tukey’s multiple comparison test) were observed in fluorescence intensity for comparison between phage treated and 24 h biofilm control, although a tendency of reduction of cell density was observed both in the quantitation panel (Fig. 6) and in the representative images (Fig. 5).

### Genomic analysis of phages demonstrates the safety potential and variability of the phages for cocktail composition

Sequencing was performed on Illumina MiSeq, and pair-end sequences (2 × 250 bases) were assembled with CLC Genomics Workbench (Qiagen). Using Trimmomatic^26^, the original reads were filtered to remove low-quality regions. Seqtk (https://github.com/lh3/seqtk) created a subsample of the total reads with 50.000 reads (one half for R1 and another half for R2). This subset of reads was used for assembly with SPAdes^27^, generating a single contig containing the whole phage genome. The contig was selected for downstream analysis.

Analysis using ABRicate with the Resfinder database (https://github.com/tseemann/abricate)^28^ showed the absence of resistance genes within the four genomes. Annotation data from RAST^29^ and PHASTER^30^ suggested that no integrase genes were found in all four genomes, and BLASTN searches with integrase genes from close phage genomes as queries also reaffirmed the absence of those genes.

**Figure 7:**
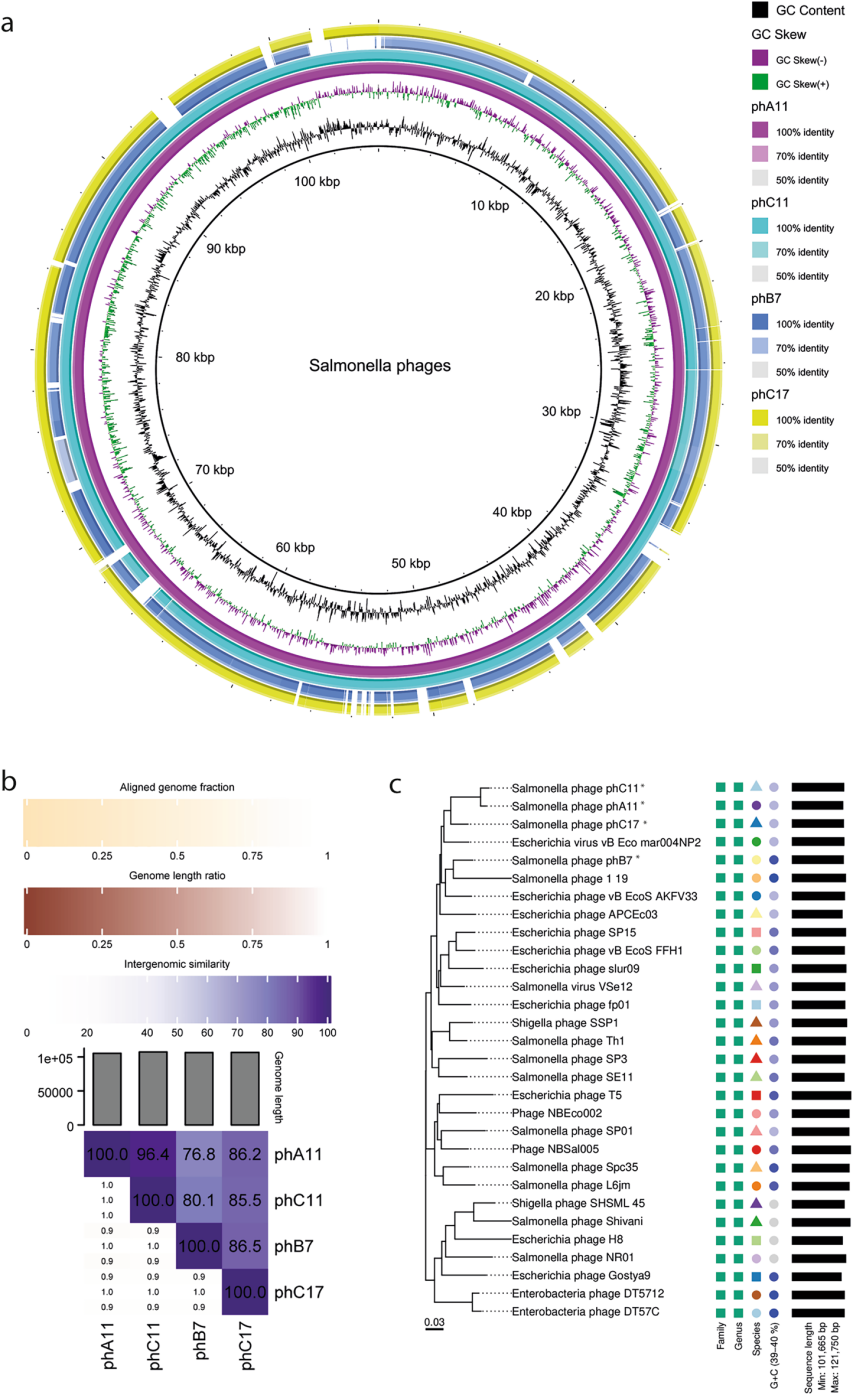
Genome analysis of phages phA11, phC11, phB7, and phC17. (**a**), Multiple genome alignment of the four phages that compose the cocktail. The genome of phA11 is used as the reference, and the alignment is a pairwise BLASTn performed using BRIG. The individual phage annotation and description are shown in Supplementary Figures S6, S7, S8, and S9. (**b**), Heatmap of intergenomic similarities and alignment indicators for the four phages of the cocktail. In the right half, color intensity is based on the intergenomic similarity of phage genomes, and the numbers represent the similarity values for each pair. In the left half, three indicator values are represented for each genome pair, in the order from top to bottom: aligned fraction genome 1 (for the genome found in the row), genome length ratio (for the genome pair), and aligned fraction genome 2 (for the genome found in the column)^32^. (**C**), Phylogenomic Genome-BLAST Distance Phylogeny (GBDP) method tree inferred using formula D0. The branch lengths of the resulting VICTOR trees are scaled in terms of the respective distance formula used. The OPTSIL clustering yielded 29 species cluster, one genus cluster, and one family cluster^34^. The shapes and colors highlight the differences and similarities in the phylogenetic classification of the phages and in the G + C content. All phages belong to the same family and genus since all have the same shape and color (green square) in the family and genus column. Regarding species, there is more diversity between the phages, where each phage is indicated as a different species.

The circular comparison of phage genomes using BRIG^31^ and Intergenomic comparisons with VIRIDIC^32^ show similarity between the four phages analyzed. Genome alignment of phC11 with phA11 demonstrates that the major genomic difference between the phages occurs between 70 and 60 kbp with small non-homologous regions in phC11’s genome (Fig. 7a). Phages phA11 and phC11 demonstrate high similarity, being assigned to the same species with 96.4% of intergenomic similarity^33^ according to the standard species threshold of 95% of VIRIDIC (Fig. 7b). Circular comparison of phB7 and phC17 genomes with phA11 genome demonstrate less similarity than phC11, with several non-homologous regions in their genomes when aligned to phA11 (Fig. 7a). Phages phB7 and phC17 have 76.8% and 86.2% of intergenomic similarity with phA11 (Fig. 7b) as demonstrated by VIRIDIC, respectively, demonstrating that although they are classified within the same genus (> 70%), they are from different species (< 95%). The intergenomic similarity between phB7 and phC17 is 86.5%, which also classifies them as different species in the same genus (Fig. 7b). This is reaffirmed by phylogeny analysis by VICTOR^34^, which grouped the four phages within the same family and genus. However, VICTOR results differentiated all the phages in different species, including phA11 and phC11, although their phylogenetic distance is low (Fig. 7c). This difference between VIRIDIC and VICTOR results regarding species level can be explained by the difference in the calculations and species thresholds used for each one. All four phages were classified in the class *Caudoviricetes*, family *Demerecviridae*, subfamily *Markadamsvirinae*, and genus *Tequintavirus*.

Holin and endolysin genes were found adjacent in all four genomes (Supplementary Figs. S6, S7, S8, and S9). Phages phA11 and phC11 endolysin and holin genes are identical. The exact similarity can be observed for phages phC17 and phB7, demonstrating that even with identical holin and endolysin genes, there are differences in the host range of the phages. Holin genes of the phages phA11/phC11 and phC17/phB7 showed 95.74% similarity, although endolysin genes showed 72.22% similarity, demonstrating more conserved regions of holin genes than endolysin genes for the four phages. BLASTn of phages phA11/phC11 endolysin genes showed high similarity (> 90%) with only five different phages, infecting *Salmonella enterica*, *Shigella sp.,* and *Escherichia coli*. For phages phC17/phB7 endolysin genes, BLASTn showed high similarity (> 90%) with seventy-five different phages, infecting *Salmonella enterica*, *Escherichia coli*, and *Klebsiella pneumoniae*.

The results of predicted tail fibers genes analysis showed that each phage had a different number of genes related to them. phA11 had eleven genes predicted as “phage tail fiber”, while phC11 had twelve genes. For phages phC17 and phB7, the number of predicted genes was ten and eight, respectively. Alignment of all these genes using Geneious version 2023.1 (Biommaters, Inc., New Zealand) demonstrated that predicted phage tail fibers of phages phA11 and phC11 share 98.38% of identity, being the most identical among the four phages. Since phage tail fibers are closely related to phage infection and, consequently, the host range, this high identity could be related to phA11 and phC11 host ranges, which are similar (Figs. 1 and 2), especially considering EOP. phB7 had the lowest identity among the four phages when considering predicted phage tail fibers, with 78.08%, 80.80%, and 81.07% for phages phA11, phC11, and phC17, respectively. Moreover, it has the lowest number of predicted phage tail fibers genes but the broadest host range on both EOP and *v*_1_. These differences may play an important role in the host range of phB7, which could be considered a generalist bacteriophage rather than a specialist one, especially in EOP analysis. Therefore, more detailed studies regarding the host range and the number and identity of phage tail fibers should be undertaken to thoroughly characterise their influence.

## Discussion

Phage therapy is considered a promising alternative to antibiotics in the fight against pathogenic bacteria, both in human medicine and animal production. Bacteriophages are extremely specific to certain bacteria; therefore, their use in a cocktail with different phages increases the host range and reduces the probability of resistance. There are numerous techniques and protocols to evaluate the lytic activity of bacteriophages, and the most used are Spot Test Assays and EOP^11,22^. For screening purposes, Spot Test is a usual technique, although it overestimates the lytic activity of the phages due to the “lysis from without” phenomenon^11,35^, which was detected by our study when comparing SST with EOP and *v*_1_. On the other side, EOP indicates productive infection, which we refer to as the ability to form plaques. It is known that the same phage could form different plaques (morphology, size, and turbidity) on different bacterial strains and that specific phage defense systems directly influence this. Several recently discovered phage defense systems like PD-λ-5, PD-λ-2, PD-T7-2, PD-T7-4, retron-Eco8, viperins, and many others, reduce the size of the plaques of phages T7, T5, T3 and λ_vir_^36–38^. Moreover, depending on the phage defense system present in the bacteria, there is a reduction in the number of plaques from ten-fold to no visible plaques, directly impacting the EOP value described^36,39^.

In infection assays in liquid culture, like *v*_1_, the presence of phage defense systems, like viperins, delay or prevent the culture collapse^38^, impacting the *v*_1_ value. Abortive infection systems like PD-T4-6^36^ and toxin-antitoxin defense systems like the recently described toxSAS CapRel subfamily^40^ directly impact the behavior of the growth curve of bacterial liquid culture infected with phages, especially at low MOIs. On the other hand, direct immunity systems do not differ in the growth curve behavior of phage-infected liquid cultures compared to uninfected control at low and high MOIs, once direct immunity systems prevent phages from multiplying without altruistic suicide behavior demonstrated by abortive infection systems^36^. Therefore, phage defense systems impact differences in the susceptibility of bacteria to the same phage when analyzed through different techniques. Phages phA11, phC11, and phC17 exhibit similar data distribution patterns in Fig. 3, in which most of the isolates have big dot size (high *v*_1_) but purple color (low EOP), demonstrating that although they possess good lytic activity against some isolates, they are not suitable “plaque formers”. A different pattern is observed for phB7, showing a high capacity to form plaques in numerous isolates, as demonstrated by a more homogenous distribution of the dot size and color. This evidence shows that the methods used could result in different classifications of the phage activity, directly impacting the choice of the phages that will compose a cocktail. It is noteworthy the difference between phages phC11 and phB7 regarding the dots patterns in Fig. 3, although they demonstrate a similar *v*_1_ average. Therefore, using only EOP scores to classify phages could lead to a sub-estimation of lytic activity, and besides that, EOP cannot detect interactions of phages in cocktails. However, this difference was not observed for phage phB7, demonstrating that depending on the phage analyzed, could be differences or not in the results and that other issues must be considered regarding the choice of the methods. Another possibility of using infection assays in liquid culture is to observe synergistic/antagonistic effects of phage combination^11^ or phage-antibiotic combinations, an important factor in designing phage cocktails.

It is important to highlight that several factors influence plaque formation. Depending on the gelling agent and its concentration, the presence of divalent cations, the growth medium used, and the growth phase of the host, the number and the size of plaques formed could be different^41^. Moreover, as stated by Abedon and Yin^41^, “plaque formation failure is not necessarily equivalent to virion inviability”, and therefore, applying more stringent criteria than the absence of plaque formation is recommended before declaring that a phage is incapable of infecting a given bacterial isolate. Considering the EOP values of phages for *Salmonella* SG, only phB7 had a high capacity to form plaques. However, all four phages could delay or control the growth of this isolate in liquid culture or even remove the biofilm during its formation when combined, as demonstrated by MTT assay and CLSM images.

Bacteria with the capacity to form biofilms reduce the efficiency of antibiotic treatments, posing a severe threat to bacterial control. Thus, searching for alternatives to antimicrobials capable of reducing biofilm formation is crucial. *Salmonella* biofilm inhibition by bacteriophages has already been described^13,42,43^. These studies demonstrated a remarkable ability to decrease the number of *Salmonella* Typhimurium and *S.* Enteritidis from biofilm structures in 96-well microtiter plates using phage cocktails at 10^7^ and 10^8^ PFU/mL. Our results demonstrated that the phage cocktail used in this study has a high capacity to control or reduce the biofilm regarding the serovar. However, it is essential to note that bacteriophages were applied to biofilms in formation, demonstrating that their prophylactic use could be efficient against biofilm formation in the food processing chain^44^. Moreover, the phages in the cocktail did not significantly (*p* > 0.05, two-way ANOVA and post hoc Tukey’s multiple comparison test) increase the growth of the biofilm for any of the tested isolates in the analysis, reinforcing the high lytic activity of the selected phages. This is of special interest since specific phages modulate several properties of their host, including the possibility of increasing biofilm production^45^. A more detailed analysis of the interaction of the phages with the *Salmonella* biofilm can be seen in the CLSM images. At 24 h of incubation, the biofilm was not mature, but initial cell clusters can be observed with high thickness. A phage cocktail applied at this stage could stop the maturation of the biofilm by reducing the cell density and preventing cell clumping after an additional 24 h of incubation since the bacterial cells were not eradicated but are dispersed, which can be inferred as a certain level of phage resistant mutant cells. The elimination of bacteria from biofilm structure by phages shows differences in effects regarding the number of phages (individual phage or phage cocktail)^46–48^, the *Salmonella* serovars, the stage of biofilm maturation, and the composition of biofilm (mixed or single species)^49^. Combining phage therapy with alternative treatments or antibiotics can be an excellent strategy to eliminate resistant mutant cells and eradicate biofilms. A recent study by Duarte et al.^50^ described the synergistic activity of a single phage and a phage-derived lytic protein against staphylococcal biofilms, demonstrating that combining phages with lytic proteins might help curtail the development of phage resistance. The combination of phage with antibiotics was also effective against biofilms, as demonstrated by Dickey and Perrot^51^, showing the prevention of antibacterial-resistant bacteria, mainly when phage was used first and antibiotic second. However, antibiotic/phage combination must be carefully studied once it could increase the distribution of antibiotic resistance genes or have an antagonistic effect, especially with drugs that act inhibiting nucleic acid or protein synthesis^52^.

Our study demonstrated that depending on the protocols used for evaluating the host range of bacteriophages, the efficacy of bacterial inhibition could be different, directly impacting the selection of phages for cocktail composition. The application of infection assays in liquid culture for host range determination, like *v*_1_, allows the evaluation of the phage cocktail effortlessly and timelessly than EOP, and therefore, it could be applied for phage cocktail design, since in EOP results, phages phA11, phC11, and phC17 did not show good plaque formation values for some *Salmonella* isolates such as the SG. However, when combined with phB7, no bacterial growth was detected in *v*_1_ and biofilm control was observed. Based solely on EOP values, these phages would not be suitable for evaluating antibiofilm activity against several *Salmonella* isolates, and this phage cocktail could be underestimated. Hence, combining strategies and techniques for better evaluation of the host range and lytic activity of bacteriophages at different conditions can demonstrate more accurately the antibacterial potential of phage cocktails. This could facilitate the use of high throughput methods and algorithms to optimize data analysis and phage combination for cocktail composition, accelerating the development process of phage therapy as the promising alternative to antibiotics.

## Methods

### Culture media and reagents

All bacteria were grown in Lysogeny-Broth (LB) Miller medium (10 g/L tryptone, 5 g/L yeast extract, and 10 g/L NaCl) or on LB Miller Agar (LBA) (1.5% agar) plates^53^. LB Miller Soft Agar (LBSA) (0.7% agar) supplemented with 10 mM CaCl_2_ was used for plaque assays. Brain–Heart Infusion broth (BHI) supplemented with 30% (v/v) glycerol was used to store bacterial isolates at -80 °C. Saline-Magnesium (SM) buffer (50 mM Tris–Cl, 100 mM NaCl, 8 mM MgSO_4_) was used for phage storage and dilution.

### Bacterial strains and growth conditions

In this study, 50 *Salmonella enterica* subsp *enterica* isolates were used (Supplementary Table S1). Standard strain ATCC 13076 was purchased from the American Type Culture Collection (ATCC, Gaithersburg, MD, United States), whereas the others were isolated from commercial poultry farms in Paraná state, Brazil, or were part of the collection of the Laboratory of Basic and Applied Bacteriology (State University of Londrina, Brazil). All bacteria were stored at -80 °C in BHI supplemented with 30% (v/v) glycerol. In each experiment, fresh 18–24 h cultures were prepared by streaking the bacteria onto LBA plates and inoculating a single colony into 5 mL of LB, incubating 18–24 h at 37 °C with shaking at 120 rpm.

### Bacteriophage isolation, propagation, and titering

Different environmental samples were collected from commercial poultry farms and slaughterhouses in Paraná state, Brazil, including poultry litter samples and slaughterhouse wastewater samples, and were used for bacteriophage isolation as described previously with some modifications^54^. In brief, liquid samples were centrifuged at 5000 × g for 10 min, and the supernatant was collected through a 0.45 µm polyethersulfone membrane filter (low protein binding membrane). For solid samples, 5 g of each sample was mixed with 10 mL of saline magnesium (SM) buffer in sterile 15 mL polypropylene centrifuge tubes and mixed thoroughly by inversion for 10 min. The tubes were centrifuged at 5000 × g for 5 min, and the supernatant was collected as the same for liquid samples. The supernatants were used for bacteriophage enrichment.

Bacteriophage enrichment was performed as described previously with some modifications^54^. Initially, 5 mL of the supernatant was mixed with 5 mL of double-strength LB supplemented with 20 mM of CaCl_2_ in 50 mL polypropylene centrifuge tubes, and 100 µL of log-phase bacterial culture was added. Polypropylene tubes with the mixture were incubated for 24 h at 37°C with shaking at 120 rpm. Then, 5% (v/v) of chloroform was added into the tubes, mixed by inversion for 5 min, and centrifuged at 5000 × g for 5 min. The supernatant was collected and stored at 4 °C. Phage activity was detected by spotting 5 µL from each supernatant onto a streak of the host strain in LBA plates supplemented with 10 mM of CaCl_2,_ and positive results were confirmed by the agar overlay method to obtain single plaques^54^. Plaques were selected based on turbidity, diameter, and appearance time. The selected plaques were removed from agar using a sterile pipette tip, inoculated into 1 mL LB supplemented with 10 mM CaCl_2_ in 2 mL microtubes, and 100 µL of log-phase bacterial culture was added. The mixtures were incubated at 37 °C for 6 h, and subsequently, phage lysates were extracted using the 5% chloroform and centrifugation procedure described above. Each lysate was titrated using the agar overlay method. This process was repeated until one plaque morphotype was obtained for each lysate.

### Determination of host range by streak spot test assay and relative efficiency of plating

The host range of isolated phages was determined against 50 *Salmonella* isolates using the Streak Spot Test (SST) method^54^, and the sensitive ones were submitted to the relative Efficiency of Plating (EOP) method^22^. Streak Spot Test was used to determine the bactericidal potential of all phages that were isolated. Phages with the broadest host range were selected to determine EOP. EOP was calculated for each phage using the bacterial isolate with maximum plaque counts versus the tested isolates, i.e., the average PFU on the tested isolate divided by the average PFU on the isolate with the maximum plaque counts. The EOP value for the phage-bacteria combination was classified as “High” for ratios ≥ 0.5, “Medium” for ratios ≥ 0.1 and < 0.5, “Low” for values > 0.001 and < 0.1, and “Inefficient” for ratios  ≤  0.001^22^. A Heatmap comparing EOP values was plotted using GraphPad Prism v. 9.1.1 (GraphPad Software).

### Dynamic lytic activity and local virulence of phages

The lytic activity in the liquid environment of selected phages was evaluated against the sensitive isolates in the Streak Spot Test assay according to Haines et al.^11^ and Storms et al.^23^ with modifications. Initially, 100 µL of double-strength LB supplemented with 10 mM CaCl_2_ was added to the flat bottom 96 well plate. Subsequently, bacteriophages were diluted to obtain 1.5 × 10^8^ PFU/mL, and 50 µL was added to each well to evaluate phage dynamic lytic activity, except in the positive (bacteria and LB broth) and negative (only LB broth) controls and blanks. The cocktail’s formulation was done by mixing each bacteriophage in a ratio of 1:1:1:1 to reach a final concentration of 1.5 × 10^8^ PFU/mL and proceed to individual phages. Overnight cultures of *Salmonella* isolates in LBA were adjusted to 0.5 MacFarland’s scale (1.5 × 10^8^ CFU/mL) in saline (0.85% NaCl), and 50 µL was added to each well, except in the negative control and blank. SM buffer was added to positive and negative controls to reach a final volume of 200 µL. Blanks had the same content as negative controls. These concentrations were used to reach a theoretical Multiplicity of Infection (MOI) of 1. The plate cover was removed and securely sealed using gas-permeable plasticized Poly-Vinyl Chloride (PVC) film. The plate was incubated in the Multiskan GO Microplate Spectrophotometer (Thermo Fisher Scientific, Vantaa, Finland), and OD(A_595nm_) readings were taken every 15 min for 12 h at 37 °C. Each test was repeated in triplicate for each *Salmonella* strain, and the data was plotted as a single killing assay curve with the mean of values using Microsoft Office Excel v. 16.0.

The area underneath the curves was calculated using the trapezoid rule for each test from 0 to 12 h. Local Virulence for MOI 1 (*v*_1_) was calculated based on the works of Storms et al.^23^ and Haines et al.^11^ by comparing the integrated area of a phage test curve (*A*_*i*_) with the integrated area of positive control (phage-free control) (*A*_0_), and the results were normalized between a theoretical minimum and maximum of 0 and 1, respectively, according to.

$$ v_{1} = 1 - \frac{{A_{i} }}{{A_{0} }} $$

Local Virulence score was classified as “High” for *v*_1_ > 0.5, “Medium” for 0.2 ≤ *v*_1_ ≤ 0.5, “Low” for 0.001 ≤ *v*_1_ < 0.2, and “Inefficient” for *v*_1_ < 0.001. A Heatmap comparing the *v*_1_ score for individual phages and the cocktail was plotted using GraphPad Prism v. 9.1.1 (GraphPad Software). R v. 4.2.2 was used to plot the comparison of EOP and *v*_1_ in a dotplot graph.

### The multiplicity of infection (MOI) assay

For the MOI assay, we assume that 100% phage adsorption had occurred soon following phage addition to bacteria^25^. Bacteriophage stocks were diluted by SM buffer into tenfold series. Bacterial host cultures (10^8^ CFU/mL) were mixed with aliquots of each dilution of the respective phage, resulting in different ratios (10^−7^ to 1 PFU/CFU) in triplicate and incubated at 37 °C for 18 h^55^. Phage lysates were titered to determine the highest production as the optimal MOI. The assay was carried out at three independent times.

### Stability of phages in different pH and temperature ranges

The stability of phages in different pH and temperatures was evaluated as described previously^56,57^ with modifications. Initially, phages were diluted in SM buffer (50 mM Tris–Cl, 100 mM NaCl, 8 mM MgSO_4_) to reach 10^8^ PFU/mL, then 100 μL of each phage was added in 900 μL of a universal pH buffer (150 mM KCl, 10 mM KH_2_PO_4_, 10 mM Na_3_C_6_H_5_O_7_, 10 mM H_3_BO_3_) with pH adjusted with NaOH or HCl from 2.5 to 12.5. A control with SM buffer (pH 7.4) was performed. The suspensions were incubated at room temperature for 24 h, and then phages were diluted and plated using the agar overlay method^58^ for enumeration. At the end of the test, the difference in titration compared to the control experiments allowed us to evaluate the viability of the phages.

To assess the stability of the phages in different temperature ranges, they were incubated in SM buffer (10^7^ PFU/mL) and stored at  − 20 °C, 4 °C (as control), 25 °C, 30 °C, 37 °C, 50 °C, 70 °C, and 90 °C for 1 h, 24 h, and 7 days (except for 50 °C, 70 °C, and 90 °C). After incubation, an aliquot of 100 μL of each phage was collected, diluted, and plated for enumeration.

### Concentration and purification of bacteriophages

Polyethylene Glycol (PEG) precipitation methodology was used with some modifications^59^ to concentrate and purify phages. A 5 mL culture of the host bacteria with the phages at the best MOI was transferred into 500 mL LB medium for culture at 37 °C overnight. Chloroform was added to the 500 mL of culture to a final concentration of 0.1% and allowed to stand at room temperature for 30 min. NaCl was added to the culture to a final concentration of 1 M, then was incubated in an ice water bath for 1 h. Subsequently, the culture was centrifuged at 11,000 × g for 10 min to remove cell debris, and polyethylene glycol 8000 (PEG8000) was added to the supernatant to a final concentration of 10% (w/v). This suspension was incubated in an ice water bath for 1 h to precipitate phage particles. The suspension was centrifuged at 11,000 × g for 10 min, and the pellet was resuspended in 5 mL of SM buffer, followed by adding 5 mL of chloroform and mixing thoroughly by inversion for 5 min and centrifuging at 5000 × g for 5 min to separate the phages from the PEG8000. Then, the aqueous phase was collected, and 3 mL of the concentrated phage suspension was centrifuged at 110,000 × g for 2 h. The supernatant was discarded, and the "glassy pellet" was resuspended in SM buffer and filtered through a 0.22 μm pore-size polyethersulfone membrane filter (low protein binding membrane) and stored at 4 °C.

### MTT biofilm assay

The phage cocktail was tested to evaluate the antibiofilm activity against *Salmonella* biofilms^60–62^. *Salmonella* samples that presented sensitivity to phages were grown in 5 mL of LB medium at 37 °C for 18–24 h. Bacterial samples were diluted 1:100 in LB medium, and 100 µL of each was added to 96 well flat bottom microtiter plates in quintuplicate for both controls and phage cocktail treated. After 24 h of incubation at 37 °C without agitation, the planktonic phase was removed, and each well was washed twice with 100 µL of pre-warmed (37 °C) PBS (137 mM NaCl, 2.7 mM KCl, 10 mM Na_2_HPO_4_, 2 mM KH_2_PO_4_). For the 24 h biofilm control, 100 µL of MTT (3-(4,5-Dimethylthiazol-2-yl)-2,5-Diphenyltetrazolium Bromide) at 0.25 mg/mL diluted in LB was added to each well and incubated at 37 °C for 2 h protected from light. After 2 h, the MTT was removed, and 100 µL of Dimethyl sulfoxide (DMSO) was added to the wells, and after 15 min of solubilization, OD readings were taken at A_570_ with the Multiskan GO Microplate Spectrophotometer (Thermo Fisher Scientific, Vantaa, Finland). For phage-treated groups, 50 µL of phage cocktail (5 × 10^7^ PFU of each phage  − 2 × 10^8^ PFU/mL of all phages) plus 50 µL of double-strength LB medium were added to the wells. For the 48 h biofilm control, only 100 µL of pre-warmed LB was added to the wells, followed by incubating the microtiter plate at 37 °C without agitation. After 24 h of incubation, the planktonic phase was removed, and each well was washed, as already mentioned, and the detection/quantification of biofilm was done accordingly previously described steps. Biofilm production was classified as negative, weak, moderate, and strong based on the cutoff value, calculated according to the following formula, using the optical density (OD) values^44^:

ODcutoff (ODc): OD average of negative control + (3 × standard deviation of ODs of negative control).

I. OD ≤ ODc = Non-producing biofilm;
II. ODc < OD ≤ (2 × ODc) = Weak biofilm producer;
III. (2 × ODc) < OD ≤ (4 × ODc) = Moderate biofilm producer;
IV. OD > 4 × ODc = Strong biofilm producer.

### Biofilm analysis by confocal scanning laser microscopy

Confocal Scanning Laser Microscopy was performed as described previously, with some modifications^50^. For confocal microscopy analysis, 24 h and 48 h biofilms were formed by inoculating 2 mL of an *S. enterica* Gallinarum (SG) cell suspension diluted 1:100 from an ON culture in LB in 24 wells plate containing a circular glass coverslip on the bottom. The plate was incubated under static conditions at 37 °C. After 24 h, the planktonic phase was removed for all groups, and the biofilm was washed twice with pre-warmed (37 °C) PBS. For the 24 h biofilm group, 400 µL of paraformaldehyde was added and incubated for 30 min at 37 °C with subsequent double washing with PBS. Next, 200 µL of 4´,6-Diamidino-2-phenylindole dihydrochloride (DAPI) was added to the wells for staining and incubated for 1 h at 37 °C. After the incubation period, the wells were washed with PBS, and the coverslip was carefully removed, added to a microscope slide with a drop of Fluoromount-G™ Mounting Medium (Sigma-Aldrich F4680), and fluorescence imaging was carried out with a TCS SP8 Confocal Scanning Laser Microscope (Leica Microsystems, Mannheim, Germany) using a 60 × water objective. The TCS SP8 confocal microscope used the LAS X Software (Leica Microsystems, Mannheim, Germany). For phage-treated groups, pre-warmed (37 °C) LB medium and phage cocktail (5 × 10^7^ PFU of each phage  − 2 × 10^8^ PFU/mL of all phages) were added to the wells and incubated for more 24 h at 37 °C. For the 48 h biofilm control, only pre-warmed (37 °C) LB was added to the wells, followed by incubation at 37 °C without agitation for 24 h. Then, the planktonic phase was removed, each well was washed as already mentioned, and the detection/quantification of biofilm was done accordingly the previously described steps.

### Extraction of bacteriophage genomic DNA

Phage DNA was extracted from highly concentrated and purified phage stock using the methodologies described elsewhere^63^ with some modifications. Purified phage particles were treated with DNase I (1 μg/mL) (Sigma-Aldrich) for 15 min at room temperature, and then the nuclease was inactivated at 75 °C for 5 min. Proteinase K (20 μg/mL) and sodium dodecyl sulfate (SDS) (1%) were then added, and the mixture was incubated at 60 °C for 1 h. To the aqueous phase, 500 µL of chloroform: isoamyl alcohol (24:1) was added and centrifuged for 5 min at 16,000 × g. The aqueous phase was precipitated with 0.8 volume times of isopropanol and centrifuged for 30 min at 16,000 × g. The pellet was washed twice with 80% ethanol, air-dried briefly, and then deionized water was used to dissolve the precipitated genomic DNA.

### Bacteriophage genome sequencing and bioinformatics analysis

Before sequencing, DNA quality and quantity were estimated using both a Qubit (Thermo Fisher Scientific) and by visualization after agarose gel electrophoresis. The library was built with the Nextera XT Illumina Kit. Sequencing was performed on Illumina MiSeq, using MiSeq Reagent 500V2. Pair-end sequences (2 × 250 bases) were assembled with CLC Genomics Workbench (Qiagen).

Quality from all the reads from sequencing was verified using FastQC^64^⁠, and its report trimming parameters were defined for Trimmomatic^26^⁠. Reads resulting from this filtering were used for genome assembly with SPAdes^27^⁠. A subset of the original reads was created using seqtk (https://github.com/lh3/seqtk), and it was used for assembly with SPAdes. Assemblies with different k-mer values were compared using QUAST^65^. The whole genome was found within a single contig selected for further analysis; other contigs were checked with BLASTN for possible fragments.

VIRIDIC^32^ was used to calculate the intergenomic similarities of the four phages, with a species threshold of 95% and a genus threshold of 70%^33^. For the circular representation of the four genomes, the software BRIG^31^⁠ was used. Features shown in supplementary images were selected via script using Biopython^66^⁠, and features identified as “Hypothetical protein” and “Phage protein” were discarded for visualization purposes.⁠

Phylogeny and classification analysis was carried out by the VICTOR web service (https://victor.dsmz.de), a method for the genome-based phylogeny and classification of prokaryotic viruses^34^. All pairwise comparisons of the nucleotide sequences were conducted using the Genome-BLAST Distance Phylogeny (GBDP) method^67^ under settings recommended for prokaryotic viruses^34^. The resulting intergenomic distances were used to infer a balanced minimum evolution tree with branch support via FASTME, including SPR postprocessing^68^ for each formula D0, D4, and D6, respectively. Branch support was inferred from 100 pseudo-bootstrap replicates each. Trees were rooted at the midpoint^69^ and visualized with ggtree^70^. Taxon boundaries at the species, genus, and family level were estimated with the OPTSIL program^71^, the recommended clustering thresholds^34^, and an *F* value (fraction of links required for cluster fusion) of 0.5^72^.

ABRicate with the Resfinder database was used to screen the genomes for the presence of genes with antimicrobial resistance and virulence factors (https://github.com/tseemann/abricate)^28^⁠. For gene calling, RAST^29^⁠ and PHASTER^30^⁠ were used. tRNA detection was carried out with tRNAscan-SE^73^. Predicted phage tail fibers genes analysis was done using the software Geneious version 2023.1 (Biommaters, Inc., New Zealand) (https://www.geneious.com).

### Accession numbers

All phage genomes were submitted to GenBank. Accession numbers for all phage genomes are OQ680478, OQ680479, OQ680480 and OQ680481 for phages phA11, phB7, phC11 and phC17, respectively.

### Statistical analysis

GraphPad Prism v.9.1.1 (GraphPad Software) was used for statistical analysis of the biofilm assays. The results were expressed as mean ± standard deviation after analysis with one-way ANOVA for DAPI fluorescence intensity data or two-way ANOVA for MTT biofilm assay data with post hoc Tukey’s multiple comparison tests for both, and *p* < 0.05 was considered significant.

### Supplementary Information

Supplementary Information 1.

Supplementary Table S1.

## Data Availability

The datasets generated during and/or analyzed during the current study are available from the corresponding author on reasonable request, and the DNA sequence of the bacteriophages are available at GenBank database under accession numbers OQ680478, OQ680479, OQ680480, and OQ680481.
