# Supplementary material for: Comparative analysis of effectiveness for phage cocktail development against multiple Salmonella serovars and its biofilm control activity
Source: Sci Rep. 2023 Aug 11;13:13054. doi: 10.1038/s41598-023-40228-z (PMC10421930; doi:10.1038/s41598-023-40228-z)
Supplement: Supplementary file 1 — Supplementary Information 1. [file 41598_2023_40228_MOESM1_ESM.pdf]

Supplementary Information

Title: Comparative analysis of effectiveness for phage cocktail development against multiple *Salmonella* serovars and its biofilm control activity

Authors

Jhonatan Macedo Ribeiro[1], Giovana Nicolete Pereira[1], Itamar Durli Junior[2], Gustavo Manoel Teixeira[3], Mariana Marques Bertozzi[4], Waldiceu A. Verri Jr[4], Renata Katsuko Takayama Kobayashi[1], Gerson Nakazato[1]*.

*Corresponding author: gnakazato@uel.br

Affiliations

[1]Laboratory of Basic and Applied Bacteriology, State University of Londrina, Londrina, Paraná, Brazil.

[2]Laboratory of Bioinformatics, Federal University of Santa Catarina, Florianópolis, Santa Catarina, Brazil.

[3]Laboratory of Microbe Biotechnology, State University of Londrina, Londrina, Paraná, Brazil.

[4]Laboratory of Pain, Inflammation, Neurology, and Cancer, State University of Londrina, Londrina, Paraná, Brazil.

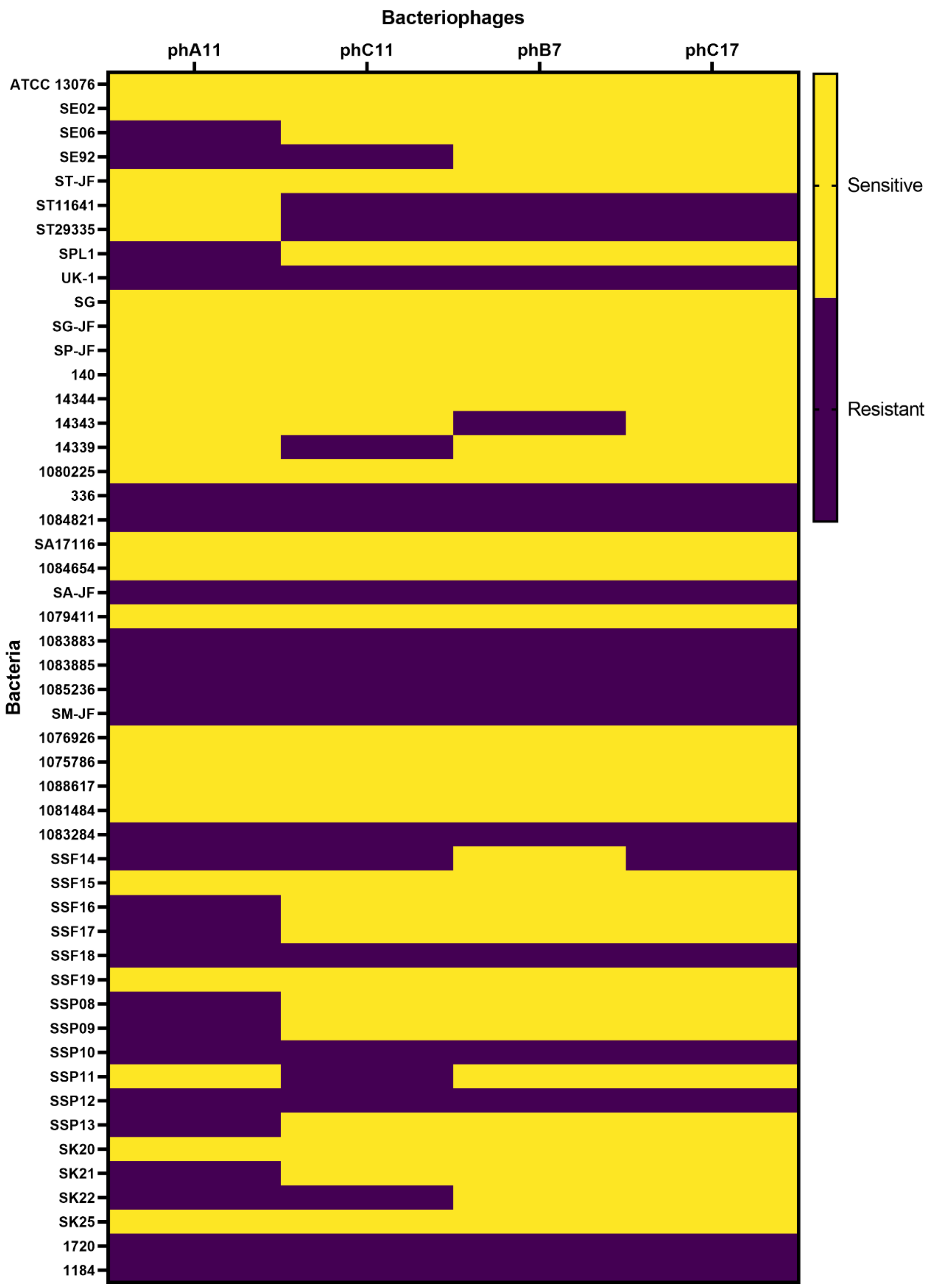

Supplementary Figure S1 - Host Range of phages phA11, phC11, phB7, and phC17 in Streak Spot Test assay.

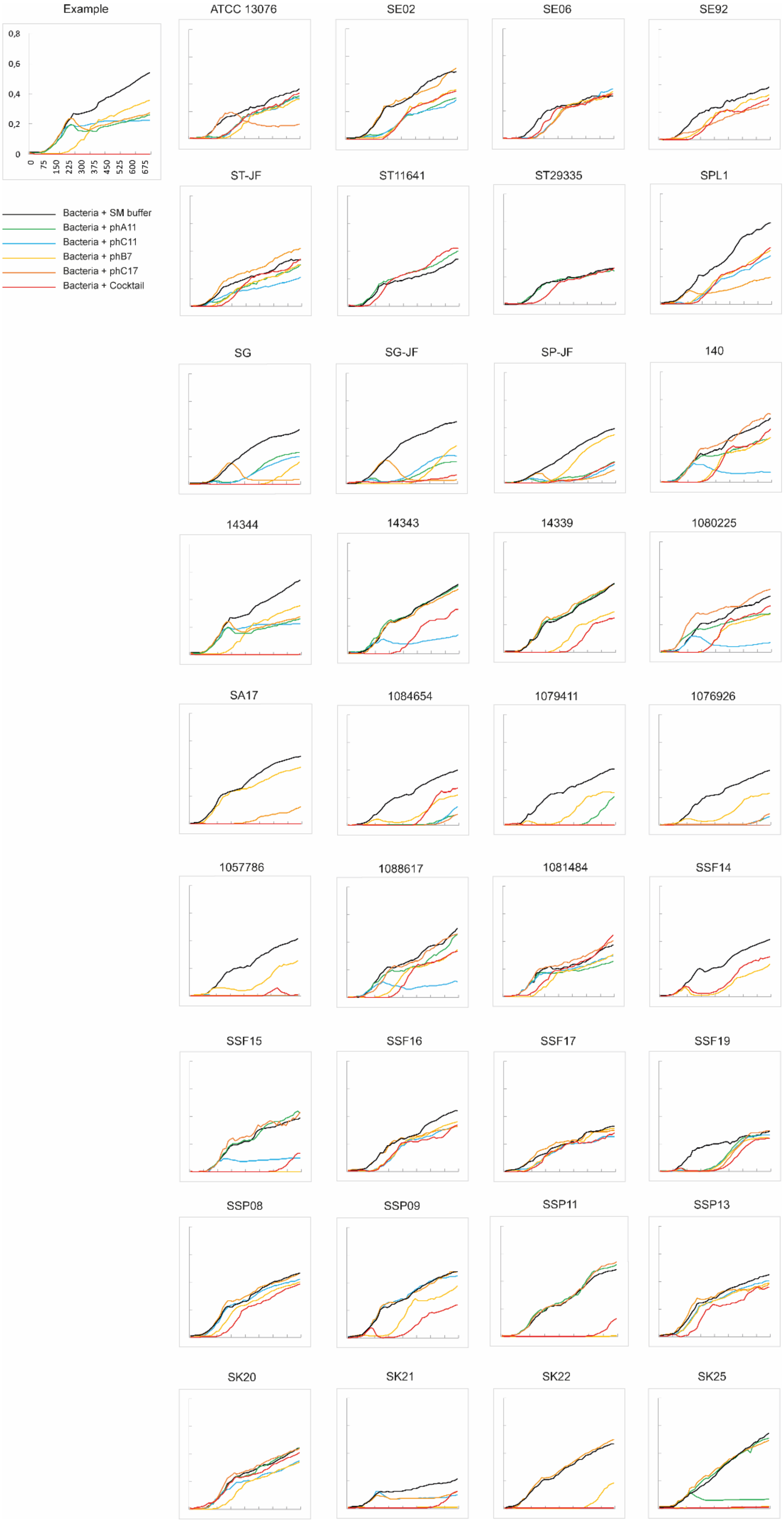

Example
0,8
0,6
0,4
0,2
0
0
75
150
225
300
375
450
525
600
675
Bacteria + SM buffer
Bacteria + phA11
Bacteria + phC11
Bacteria + phB7
Bacteria + phC17
Bacteria + Cocktail
ATCC 13076
SE02
SE06
SE92
ST-JF
ST11641
ST29335
SPL1
SG
SG-JF
SP-JF
140
14344
14343
14339
1080225
SA17
1084654
1079411
1076926
1057786
1088617
1081484
SSF14
SSF15
SSF16
SSF17
SSF19
SSP08
SSP09
SSP11
SSP13
SK20
SK21
SK22
SK25

Supplementary Figure S2 - Killing assay curves of 36 *Salmonella* isolates.

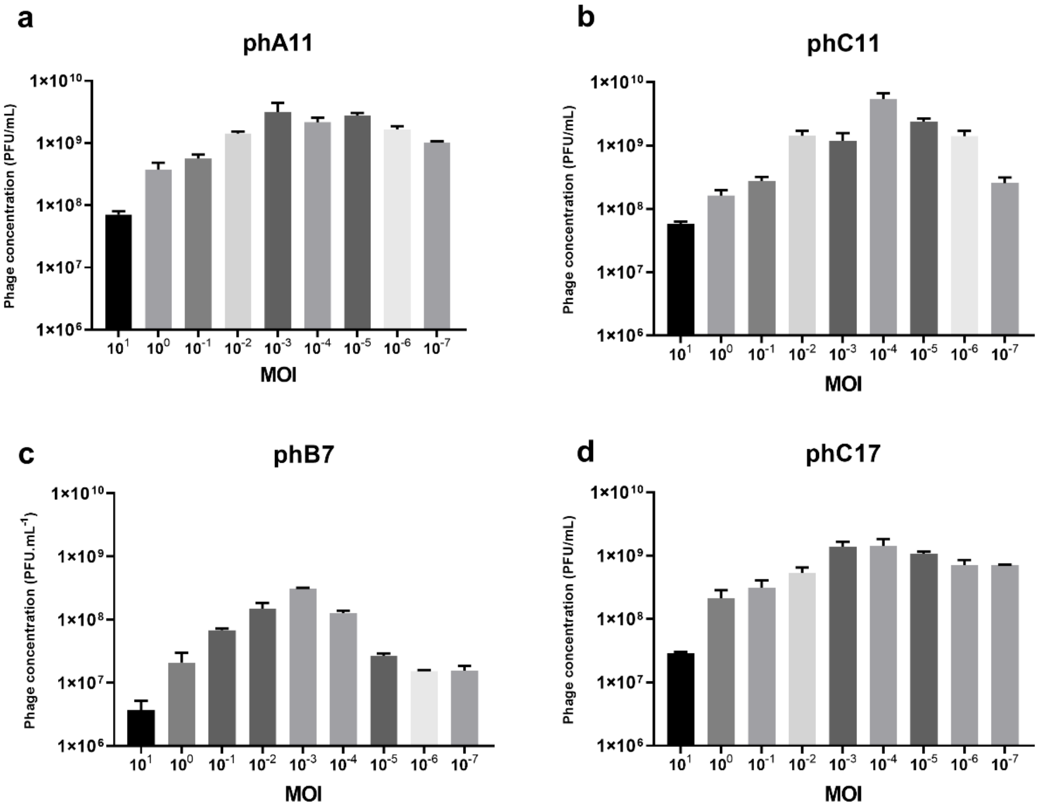

Supplementary Figure S3 - Bacteriophage production at different Multiplicity of Infection for phages (**a**) phA11, (**b**) phC11, (**c**) phB7, and (**d**) phC17.

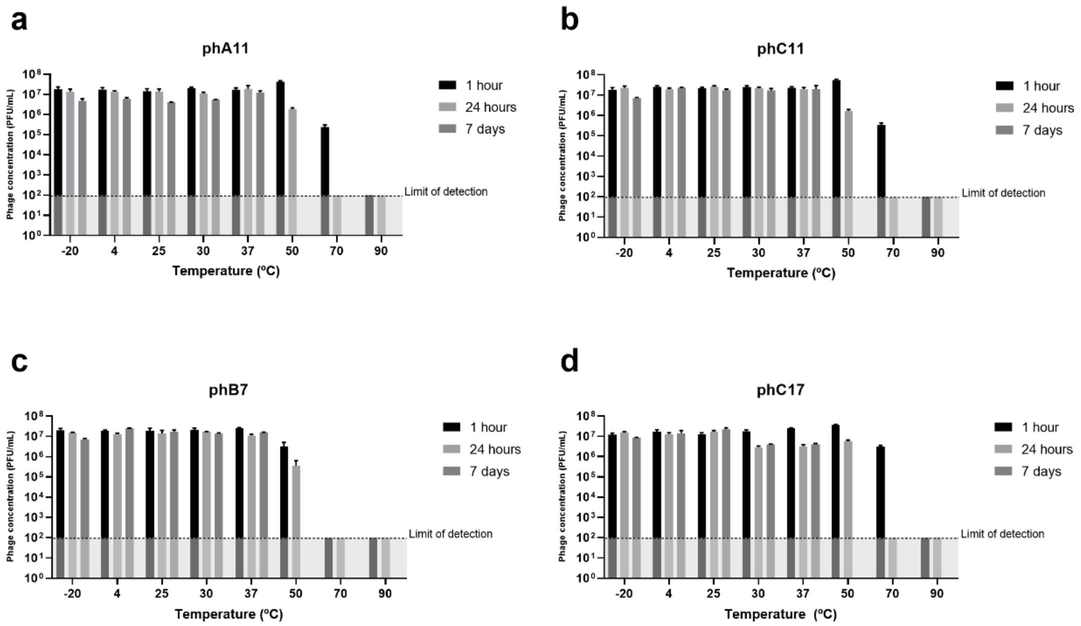

Supplementary Figure S4 - Temperature resistance of phages (**a**) phA11, (**b**) phC11, (**c**) phB7, and (**d**) phC17.

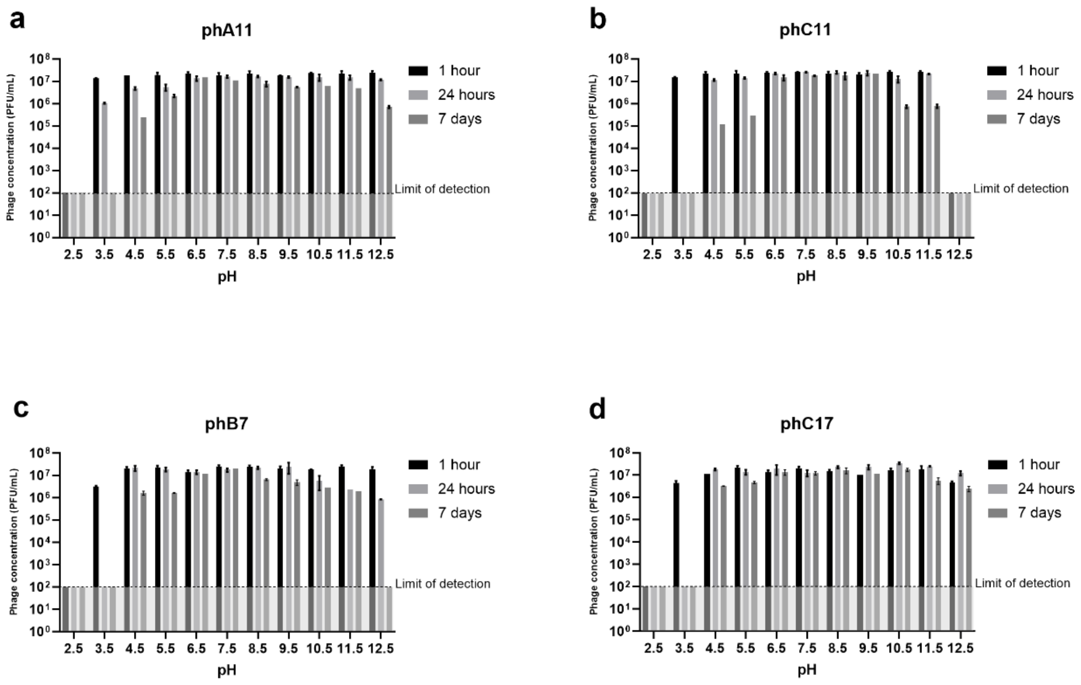

Supplementary Figure S5 - pH resistance of phages (**a**) phA11, (**b**) phC11, (**c**) phB7, and (**d**) phC17.

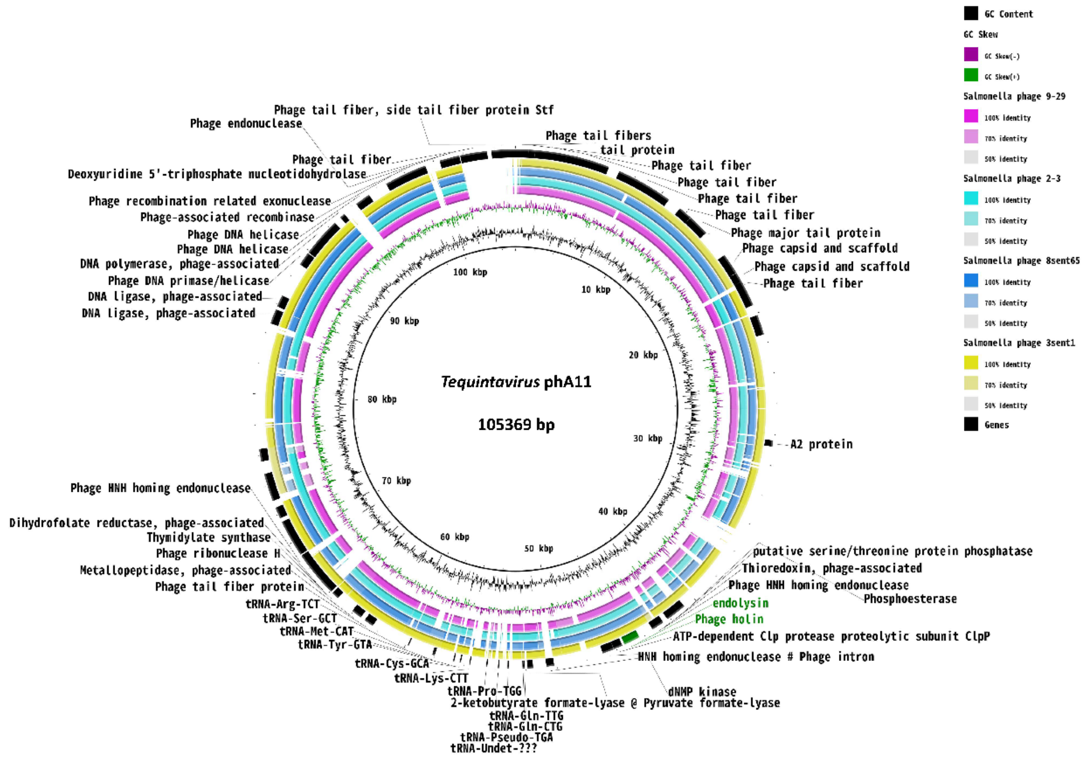

Supplementary Figure S6 - Genome annotation and alignment of phage *Tequintavirus* phA11.

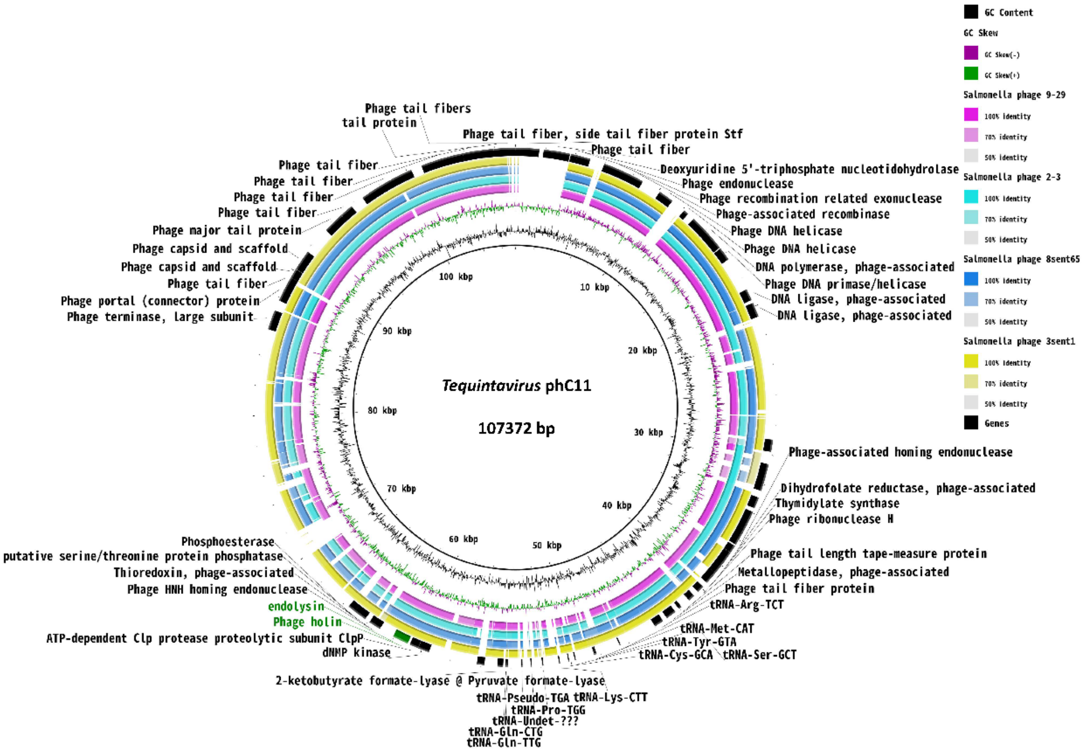

Supplementary Figure S7 - Genome annotation and alignment of phage *Tequintavirus* phC11.

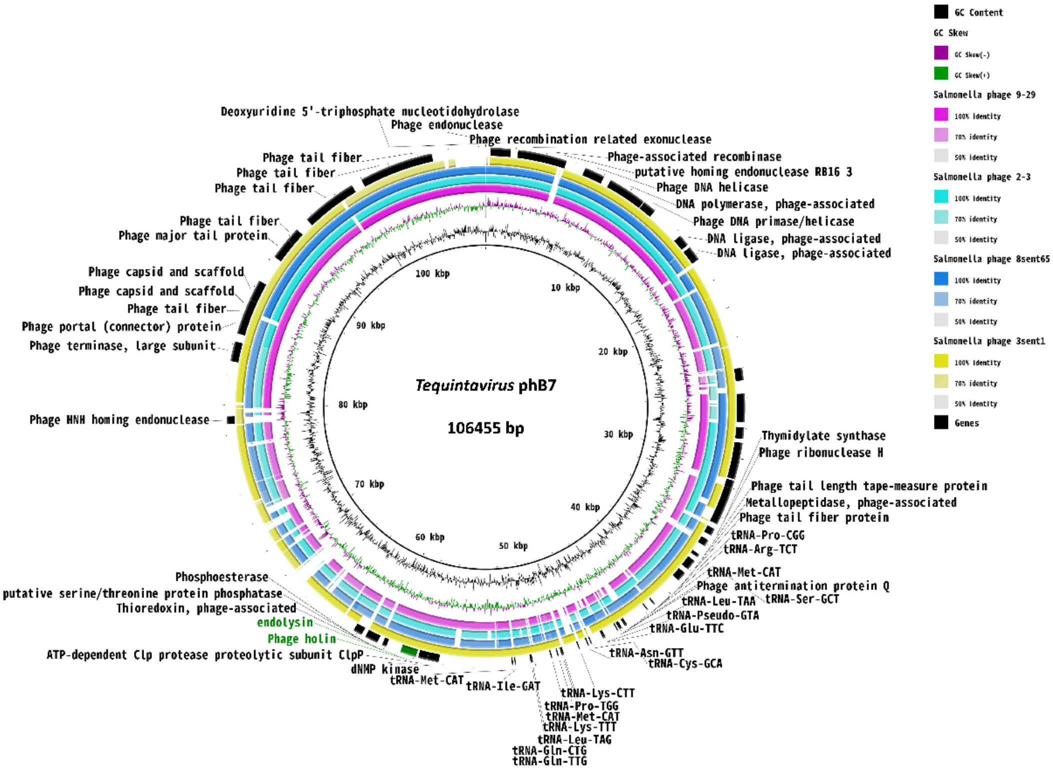

Supplementary Figure S8 - Genome annotation and alignment of phage *Tequintavirus* phB7.

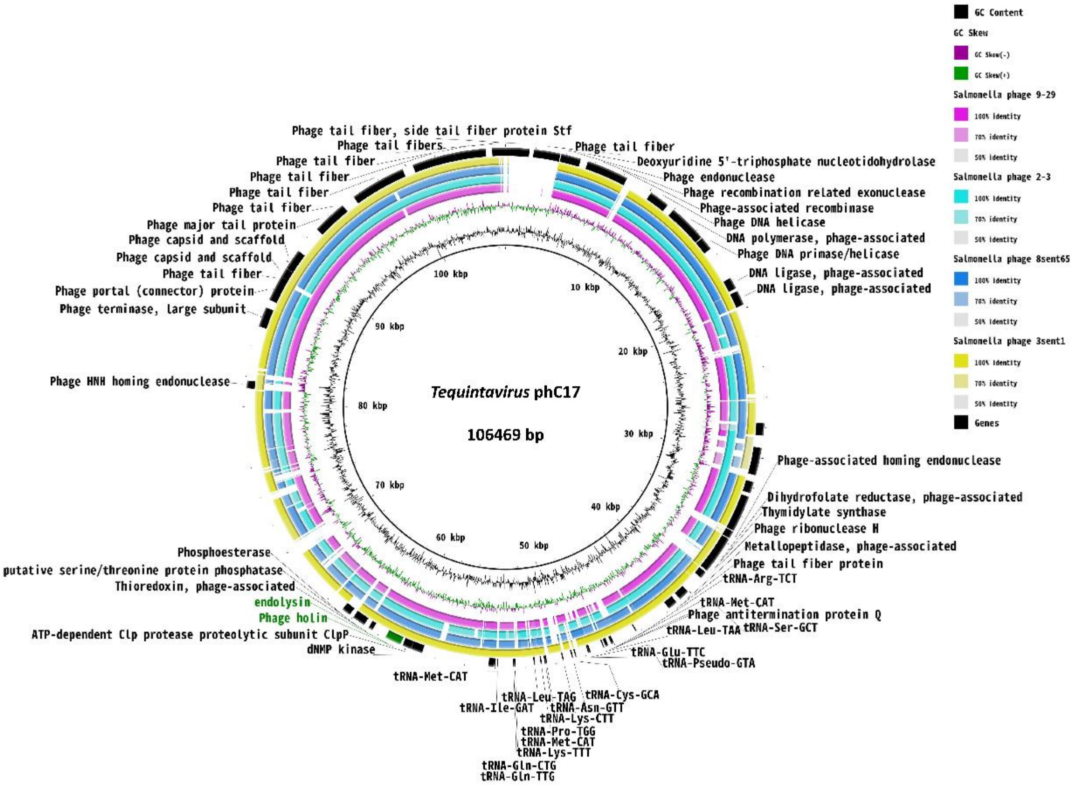

Supplementary Figure S9 - Genome annotation and alignment of phage *Tequintavirus* phC17.
